# Differential Antitumoral Properties and Renal-Associated Tissue Damage Induced by Tacrolimus and Mammalian Target of Rapamycin Inhibitors in Hepatocarcinoma: In Vitro and In Vivo Studies

**DOI:** 10.1371/journal.pone.0160979

**Published:** 2016-08-12

**Authors:** Elena Navarro-Villarán, José Tinoco, Granada Jiménez, Sheila Pereira, Jize Wang, Sara Aliseda, María A. Rodríguez-Hernández, Raúl González, Luís M. Marín-Gómez, Miguel A. Gómez-Bravo, Francisco J. Padillo, José M. Álamo-Martínez, Jordi Muntané

**Affiliations:** 1 Institute of Biomedicine of Seville (IBIS), “Virgen del Rocío”-“Virgen Macarena” University Hospital/CSIC/University of Seville, Seville, Spain; 2 Department of General Surgery, “Virgen del Rocío”-“Virgen Macarena” University Hospital/CSIC/University of Seville/IBIS/CSIC/University of Seville, Seville, Spain; 3 Centro de Investigación Biomédica en Red de Enfermedades Hepáticas y Digestivas (CIBERehd), Madrid, Spain; University of Navarra School of Medicine and Center for Applied Medical Research (CIMA), SPAIN

## Abstract

Orthotopic liver transplantation (OLT) is the recommended treatment for patients at early stages of hepatocarcinoma (HCC) with potential portal hypertension and/or bilirubinemia, but without vascular-associated diseases. The patients are receiving immunosuppressive therapy to reduce graft rejection, but differential side effects have been related to calcineurin and mTOR inhibitor administration regarding tumor recurrence and nephrotoxicity. The *in vitro* studies showed that Tacrolimus exerted a more potent pro-apoptotic effect than Everolimus (Huh 7>Hep 3B>HepG2), being sirolimus only active in Hep3B cell line. Tacrolimus and Everolimus exerted potent antiproliferative properties in Huh 7 and Hep3B in which cells Sirolimus was inactive. Interestingly, Tacrolimus- and Everolimus-dependent G_0_/G_1_ cell accumulation occurred as a consequence of drastic reduction in S, as well as in S and G_2+M_ phases, respectively. The in vivo studies support data on the more effective antitumoral properties of Everolimus, eventual risk of pro-angiogenic tumoral properties and nephrotoxicity of Tacrolimus, and pro-proliferative properties of Sirolimus in tumors developed in nude mice.

## Introduction

Hepatocellular carcinoma (HCC) is the fifth most common neoplasia in the world, and the third most common cause of cancer-related mortality worldwide (600,000 deaths per year) [1–3]. HCC is the main primary malignancy in the liver causing death in cirrhotic patients [4]. The performance status and hepatic function of the patient, number and size of the nodules, tumor vascular invasion, and the presence of extrahepatic metastasis, is actually used for the staging, prognosis as well as the therapeutic recommendation to the patient with HCC [5]. The curative treatments (ablation, resection and orthotopic liver transplantation or OLT) are indicated at the very early stage (Barcelona Clinic Liver Cancer or BCLC 0) and at the early stage (BCLC A) of the disease characterized by the presence 1–3 tumors less than or equal to 3 cm, good liver function (Child-Pugh A-B), asymptomatic (Performance Status or PS 0), and absence of vascular invasion and extrahepatic metastases. OLT is indicated in patients with potential portal hypertension and/or bilirubinemia, but without vascular-associated diseases [5]. The patients are receiving immunosuppressive therapy to reduce graft rejection. The mechanisms by which immunosuppressants exert their effects are different. Cyclosporine and Tacrolimus bind to respective cyclosporine A binding proteins (cyclophilins or CyPs) and immunophilin FK506-binding protein (FKBP), resulting in the prevention of calcium/calmodulin-dependent calcineurin-related dephosphorylation of the nuclear factor of activated T cells (NFAT) that drives upregulation of IL-2 production in T cells, and thereby attenuating cytokine receptor-dependent mammalian target of rapamycin (mTOR) activation and lymphocyte proliferation [6]. FK506 also antagonizes the interaction of another transcription factor, cAMP response element-binding protein (CREB) with its putative DNA binding site, CRE, which in turn inhibited cAMP-directed transcriptional events [7]. The molecular mechanism of action of mTOR inhibitors, Everolimus and Sirolimus, is based on the binding to the immunophilin FKBP12, which resulting complex reduces mTOR-1-dependent p70S6K1 and 4E-BP1 activation that regulates protein translation and cell cycle progression [8]. mTOR inhibitors downregulate translation affecting protein expression involved in cell cycle progression such as cyclin D1, c-Myc, p21, as well as apoptosis prevention such as Bcl-XL [9, 10]. In the context of immune system, mTOR inhibitors prevent the proliferation and clonal expansion of antigen-activated T-cells. Over the past few years, additional members of the FKBP and NFAT families of proteins have been identified, providing further insights into the complexity of cell signaling that may account for the adverse side effects of the drug, including neurotoxicity and nephrotoxicity [11].

The conventional immunosuppressant drugs or calcineurin inhibitors (CNI) (Cyclosporine and Tacrolimus) have been associated with a dose-dependent increase in the risk of tumor recurrence after OLT [12, 13], compared with mTOR inhibitors-based immunosuppression (Sirolimus and Everolimus) which have been associated with increased survival of patients undergoing OLT for HCC [14, 15]. The objective of the present study was to evaluate in vitro and in vivo the differential pro-apoptotic and anti-proliferative properties of Tacrolimus and mTOR inhibitors, and their correlation to nephrotoxicity in an experimental xenograft mice model.

## Material and Methods

### Drugs

Everolimus (Certican^®^, Novartis, Basilea, Switzerland), Sirolimus (Rapamune®, Pfizer, New York, USA) and Tacrolimus (Prograf, Astellas Pharma Inc., Tokio, Japan) were solved in DMSO (95.8, 91.5 and 80.4 μg/μl, respectively) in order to obtain working solution useful for the in *vitro* experiments. The drugs were diluted in ethanol (1 μg/μl) in order to obtain working solution useful for the in vivo experiments.

### Cell lines and culture conditions

HepG2 and Hep3B were obtained from American Type Culture Collection (ATCC; LGC Standards, S.L.U., Barcelona, Spain). Huh 7 was purchased from Apath, LLC (Brooklyn, NY, USA). Cell lines were selected according to p53 content: HepG2 (wt p53 expression), Huh 7 (p53 mutated isoform, codon 220), and Hep3B (no-sense p53 mutation). All cell lines were negative for mycoplasma contamination. Cells were cultured in MEM with Earle’s salts with L-glutamine (Ref E15-825, PAA) with 10% FBS (F7524, Sigma-Aldrich, Lot No: 022M3395, endotoxin <0.2 EU/ml), sodium pyruvate (1 mM) (Ref S11-003, PAA), non-essential amino acids (Ref M11-003, PAA), Penicillin-Streptomycin solution (100 U/mL-100 μg/ml) (P11-010, PAA), at 37°C in a humidified incubator with 5% CO_2_. Cells were cultured at cell density 100,000 cells/cm^2^. Cell confluence was never reached. The treatments were added 24 after plating. The effect of immunosuppressants were administrated at a broad range of concentrations (0, 10 nM, 100 nM, 1 μM, 10 μM and 100 μM). Cell lysate was obtained at 12 and 24 hours after drug administration.

### In vivo experimental model

Twenty-four male Hsd:Athymic Nude-Foxn1nu (Harlan Laboratories, Barcelona, Spain) aged 4 weeks (weighing 18–22 g) were randomly distributed in four groups: Control, Everolimus, Sirolimus and Tacrolimus. Everolimus (0.02 mg/g per day, oral administration), Sirolimus (0.04 mg/g per day, oral administration) and Tacrolimus (0.02 mg/g per day, oral administration) solutions were prepared diluting a volume of (20, 40 and 80 μl stock solution, respectively) in 8 ml 5% glucose. Control animals received a volume of 80 μl stock solution in 8 ml 5% glucose.

Huh 7 (6x10^6^/50 μl culture minimum eagle medium) were diluted in 50 μl of matrigel (Ref 356231, Becton-Dickinson, Lot 36821, 9.8 mg protein/ml) and administered subcutaneously into the right flank of mice. The treatments were administered 24 h after tumor cell implantation. The animals of each experimental group were monitored each 48 hours to recognize pain, distress and discomfort in experimental animals caused by the treatments following the supervision protocol described by Morton and Griffiths [16]. Any symptoms of the described by the supervision protocol were observed before the experimental endpoint. Animals were sacrificed under ketamine and diazepam anesthesia when tumor from one animal reached 15 mm following the criteria for experimental cancer models described by Workman et al [17]. Tumors were obtained in sterile conditions and their volume were calculated using the formula: V = [(length)x(width)x(depth)xπ]/6. Tumors were excised in small fragments (2 mm^3^) and fixed in 4% paraformaldehyde. No tumors were observed in peritoneum, liver and lungs. All animal care and experimentation conformed to the Guide for the Care and Use of Laboratory Animals published by the National Academy of Sciences. The Bioethical Committee of the Institution gave the approval of the experimental procedure.

### Detection of ^Ser2481^mTOR and mTOR in HepG2 cells

The expression of ^Ser2481^mTOR and mTOR was analyzed in HepG2 by Western-Blot analysis. Cells were treated with lysis buffer containing 50 mM HEPES pH 7.5, 5 mM EDTA, 150 mM NaCl, 1% NP-40, 0.5 mM PMSF, 1 mM DTT, 1 mM NaF, 1 mM Na_3_VO_4_ and commercial protease inhibitor solution (Sigma, P8340) for 20 min on ice with regular vortexing each 5 min followed by centrifugation of the sample at 15,000 x g for 5 min at 4°C. The supernatant (total cell lysate) were stored at –80°C until use. Proteins (50 μg) were loaded and separated onto any-kD 18-well CriterionTM TGX Stain-FreeTM precast SDS-polyacrylamide gels (Bio-Rad Laboratories, Hercules, CA, USA) at 300 V 20–25 min. The gels were then activated by exposure to UV light for 1 min to visualize the proteins using the ChemiDocTM MP System. Proteins were transferred to PVDF membrane blot in 10 min using the Trans-Blot® TurboTM transfer system. A stain-free blot image was taken using the ChemiDocTM MP System for total protein measurement in each sample lane. After blocking with commercial solution, the blots were incubated using commercial polyclonal primary antibodies against ^Ser2481^mTOR (#2974, Cell Signaling Technology, Inc., Danvers, MA, USA) and mTOR (#2983, Cell Signaling Technology Inc.) and the corresponding secondary antibodies labeled with horseradish peroxidase (sc-2031, Santa Cruz Biotechnology) revealing protein content by ECL. Chemiluminiscence was measured using an Infinite 200 PRO Microplate Reader (TECAN Group Limited, Männedorf, Suiza).

### Measurement of cell death

Caspase-3-associated activity was determined using Caspase-Glo® 3 Assay Systems (G8091, Promega, Fitchburg, Wisconsin, USA). Cells were treated with Caspase-Glo® 3 Reagent in an "add-mix-measure" format resulting in cell lysis, caspase-3-dependent cleavage of the substrate and generation of a "glow-type" luminescent signal. The signal generated is proportional to the amount of caspase-3 activity. The values are extrapolated into a calibration curve included in the assay. Chemiluminiscence was measured using an Infinite 200 PRO Microplate Reader (TECAN).

Apoptosis was also determined in fixed cultured cells, as well as in tumor and renal tissue sections using the DeadEnd™ Fluorometric TUNEL System (Promega, G3250). The DeadEnd™ Fluorometric TUNEL System measures nuclear DNA fragmentation, an important biochemical hallmark of apoptosis in many cell types. The DeadEnd™ Fluorometric TUNEL System measures the fragmented DNA of apoptotic cells by catalytically incorporating fluorescein-12-dUTP at 3´-OH DNA ends using the enzyme Terminal Deoxynucleotidyl Transferase (TdT), which forms a polymeric tail using the principle of the TUNEL (TdT-mediated dUTP Nick-End Labeling) assay. The fluorescein-12-dUTP-labeled DNA was visualized using Olympus BX61 microscope. Fluorescence quantification was performed using Leica Application Suite Advanced Fluorescence software and ImageJ software.

### Cell proliferation

The measurement of bromodeoxyuridine (BrdU) incorporation was used as marker of cell proliferation (Roche Diagnostics, Ref. 11 647 229 001, Mannheim, Germany). Cells were seeded at low density (12,500 cells/cm2) at 37°C in a humidified incubator with 5% CO_2_. After 24 h of stabilization cells were treated with drugs including corresponding controls without BrdU. Two hours before evaluation of cell proliferation (12 h) 20 μl of BrdU (10 μM) was added to culture. DNA was denaturalized with 200 μl FixDenat solution included in the commercial assay for 30 min a room temperature. After removal, cells were incubated with 100 μl monoclonal anti-BrdU antibodies conjugated with horseradish peroxidase for 90 min at room temperature. Afterwards, cells were washed with phosphate buffer saline (PBS) (137 mM NaCl, 2.7 mM KCl, 4.3 mM Na_2_HPO_4_), and incubated with 100 μl reveling solution including hydrogen peroxide, luminol and 4-iodophenol for 15 min at room temperature. Absorbance was measured at 370 nm using an Infinite 200 PRO Microplate Reader (TECAN).

Cell proliferation was also determined by the procedure described by Darzynkiewicz et al. [18]. Briefly, cells recovered using enzymatic cell dissociation solution where washed twice with PBS supplemented with 0.1% BSA and 5 mM EDTA pH 7.4 at 50 *g* for 5 min. Cells (10^6^ cells/ml) was resuspended in ethanol 70% and kept at -20°C. Further incubation of hepatocytes with ethanol 70% for 4 h at 4°C allowed cell permeabilization. Cells were washed twice with 0.990 ml PBS supplemented with 0.1% BSA and 5 mM EDTA pH 7.4, and incubated with RNasa A (5 U/ml, 5 μl Stock 2) and with propidium iodide (20 μg/ml, 5 μl Stock 2) for 10 min. DNA content of hepatocytes was evaluated by the emission of fluorescence from the DNA-propidium iodide complex detected by a BD FACS Canto II using el BD FACSDiva Software (Becton-Dickinson, San Jose, CA). Distribution of cells, either in apoptosis or in cell cycle, in relation to DNA content was determined using an application of the flow cytometer. A double-discriminator module was used to distinguish between signals coming from a single cell or from those produced by cell aggregates.

### Histological analysis

Tumor and renal fixed tissue sections (5 μ) were obtained to analyze tissue structure and fibrosis using classical hematoxylin-eosin and Masson staining, as well as to analyze the degree of angiogenesis, fibrogenesis and cell proliferation using the expression of CD31, α-smooth muscle actin (alpha-sma) and Ki67 by immunohistochemistry, respectively. Sections were deparaffinised, hydrated through graded ethanol steps, briefly rinsed in water and blocked at room temperature using TBSA-BSAT (10 mM Tris, 0.9% NaCl, 0.02% sodium azide, 2% bovine serum albumin and 0.1% Triton-x100 detergent). Slices were incubated overnight at room temperature with primary antibodies against 1:50 CD31 (Abcam, ab28364), 1:250 alpha-sma (Abcam, ab5694) and 1:500 Ki67 (DAKO, IR626) followed by incubation with the corresponding secondary antibodies either Alexa 488 Anti-rabbitt IgG (Invitrogen, A11008) or Alexa 488 Anti-mouse IgG (Invitrogen, A11001) for 5 hours diluted in TBSA-BSAT (1:500). Nuclear staining was performed using DRAQ-5^th^ (Red Fluorescen Cell-Permeable DNA probe, Biostatus Limited, United Kingdom). Immunofluorescence analysis was performed using Olympus BX61 microscope. Fluorescence quantification was performed using Leica Application Suite Advanced Fluorescence software and ImageJ software.

## Results

### Differential pro-apoptotic properties of Tacrolimus and mTOR inhibitors in differentiated HCC cells

**Fig 1:**
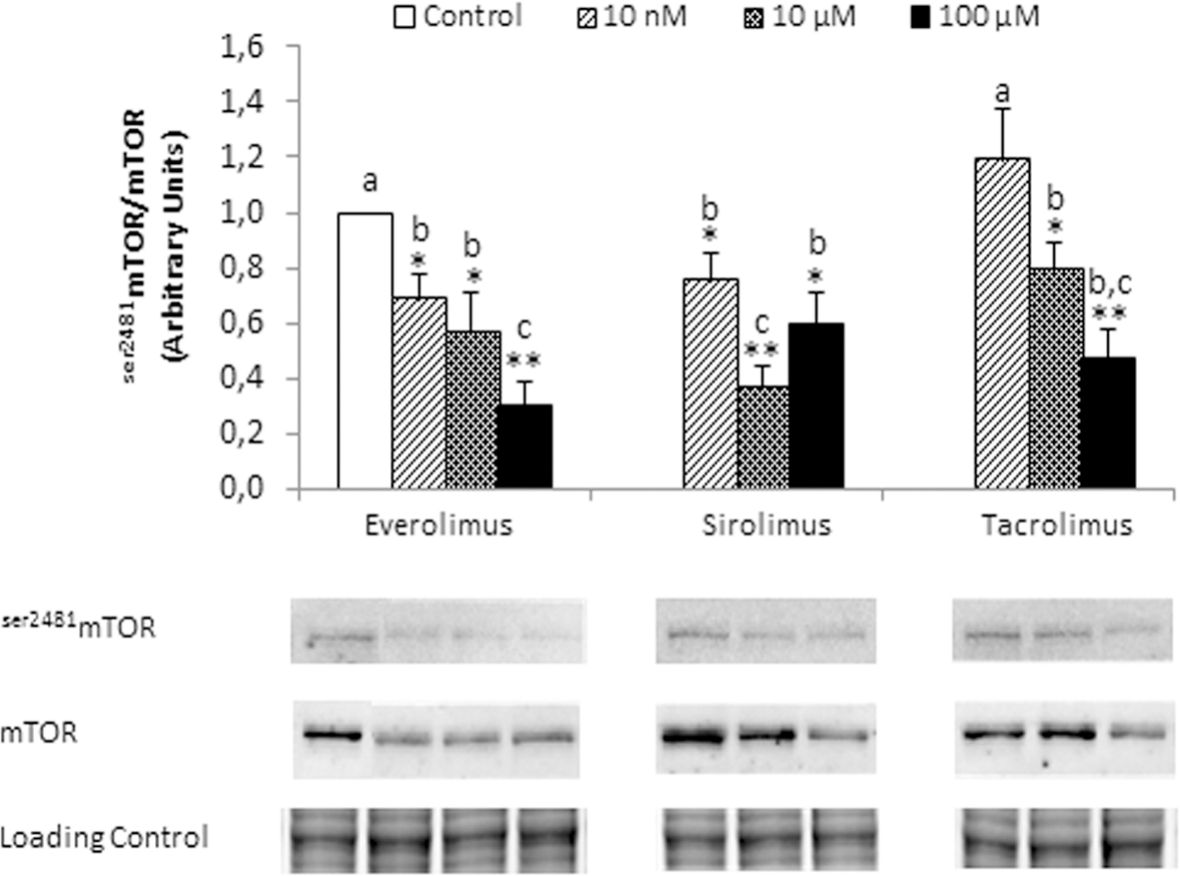
^Ser2481^mTOR/mTOR ratio in HepG2 treated with Everolimus, Sirolimus and Tacrolimus. The expression of ^Ser2481^mTOR and mTOR was assessed at 6 hours after treatments (0, 10 nM, 10 μM and 100 μM) by Western-blot analysis. Data are expressed as mean ± SEM. The groups with symbol are statistically different (*p≤0.05 or **p≤0.01) compared with their corresponding control. The groups with different letter (a, b or c) were significantly different (*p* ≤ 0.05) compared to other groups. The images are representative of four independent experiments.

**Fig 2:**
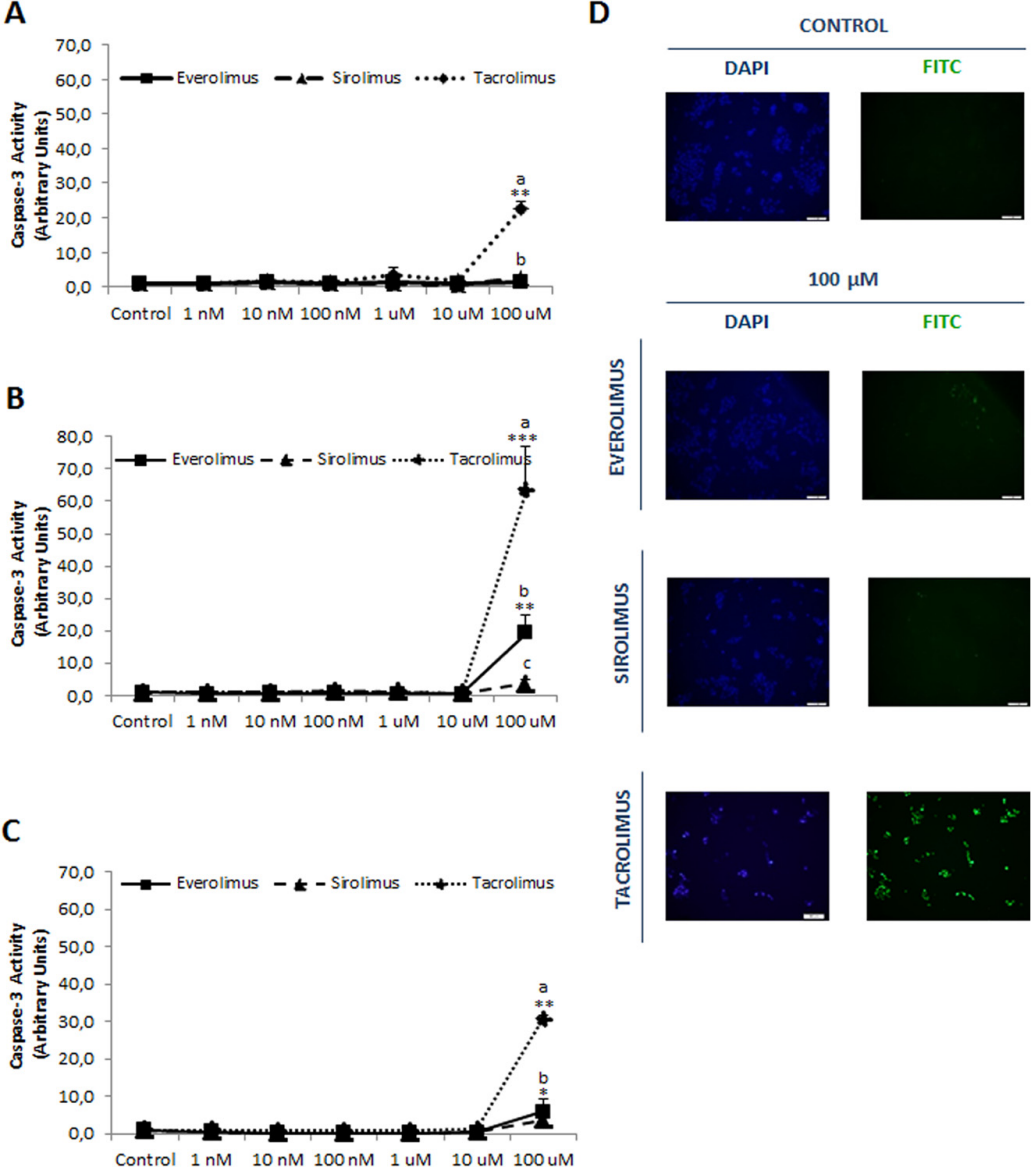
Caspase-3 associated activity in HepG2 (A), Huh 7 (B) and Hep3B (C) treated with Everolimus, Sirolimus and Tacrolimus. TUNEL staining in control and immunosuppressant-treated Huh 7 cells (D). The enzymatic activity has been assessed by fluorimetric commercial assay at 24 h after treatment (0, 10 nM, 100 nM, 1 μM, 10 μM and 100 μM). TUNEL was determined at 24 h after treatments (100 μM) using commercial assay. The induction of caspase-3 and TUNEL was only detected at the highest drug concentration. Data are expressed as mean ± SEM. The groups with symbol are statistically different (*p≤0.05, **p≤0.01 or ***p≤0.001) compared with their corresponding control. The groups with different letter (a, b or c) were significantly different (*p* ≤ 0.05) compared to other groups within the same drug concentration. The images are representative of four independent experiments.

The functional confirmation of mTOR inhibition, such as ^Ser2481^mTOR/mTOR ratio, by all treatments is shown in Fig 1. The induction of apoptosis by immunosupressants has been determined measuring caspase-3 and TUNEL-staining in differentiated HCC cell lines. Tacrolimus (100 μM) induced apoptosis in HepG2 (Fig 2A, *p*≥0.01), Huh 7 (Fig 2B, *p*≥0.001) and Hep3B (Fig 2C, *p*≥0.01). Everolimus (100 μM) induced caspase-3 in Huh 7 (Fig 2B, *p*≥0.01) and Hep3B (Fig 2C, *p*≥0.05), but Sirolimus only induced caspase-3 in Hep3B (Fig 2C, *p*≥0.05). Interestingly, HCC cell lines have differential susceptible to the apoptotic properties of immunosuppressant (Huh 7>Hep3B>HepG2), being HepG2 cells resistant to the pro-apoptotic properties of Everolimus and Sirolimus (Fig 2). The induction of apoptosis of Tacrolimus and Everolimus was confirmed in TUNEL staining in Huh 7 (Fig 2D).

### Differential anti-proliferative properties of Tacrolimus and mTOR inhibitors in differentiated HCC cells

**Fig 3:**
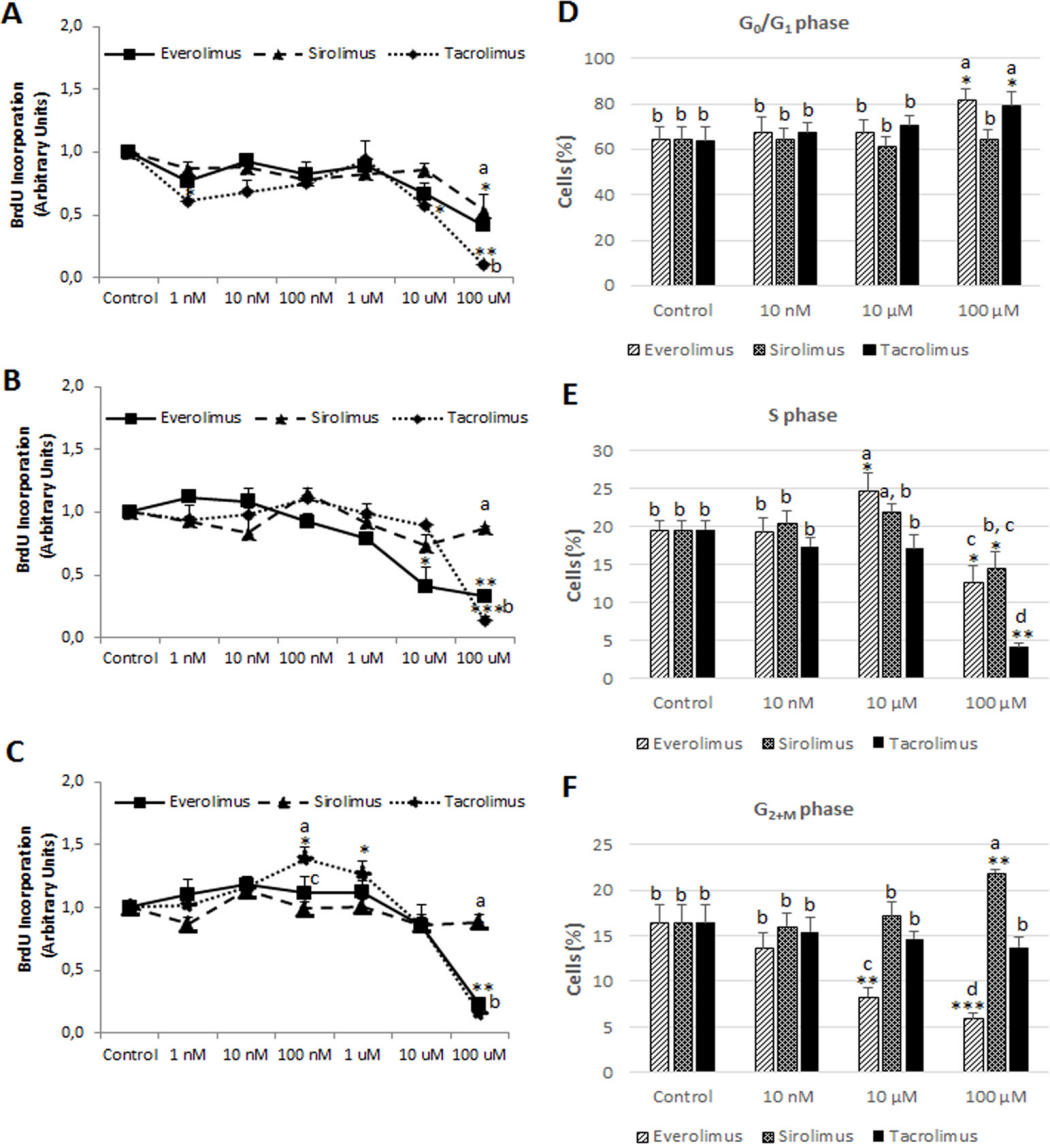
Cell proliferation in HepG2 (A), Huh 7 (B) and Hep3B (C) treated with Everolimus, Sirolimus and Tacrolimus. Measurement of the percentage of cells distributed in G_0_/G_1_ (D), S (E) and G_2+M_ (F) phases of cell cycle in HepG2 cells. Cell proliferation was determined by bromodeoxyuridine (BrdU) incorporation (A-C) and cell cycle distribution by DNA staining using propidium iodide (D-F) as described in Material and Methods. Cell proliferation was determined at 12 h after treatment (0, 10 nM, 100 nM, 1 μM, 10 μM and 100 μM). Data are expressed as mean ± SEM. The groups with symbol are statistically different (*p≤0.05, **p≤0.01 or ***p≤0.001) compared with their corresponding control. The groups with different letter (a, b, c or d) were significantly different (*p* ≤ 0.05) compared to other groups within the same drug concentration.

Tacrolimus and Everolimus exerted a significant more potent antiproliferative properties in comparison with Sirolimus in Huh 7 (Fig 3B) and Hep3B (Fig 3C) cells (*p*≥0.01). The antiproliferative activity of Sirolimus was only significant and at similar extent than Everolimus in HepG2 (*p*≥0.05), being Huh 7 and Hep3B cell lines resistant to the regulation of cell proliferation by Sirolimus. Interestingly, the intermediate concentration ranges of Tacrolimus (100 nM-1 μM) increased cell proliferation in Hep3B (Fig 3C, *p*≥0.05). The measurement of the percentage of cells distributed in each cell cycle phases (G_0_/G_1_, S and G_2+M_) was carried out in HepG2 cells that respond to all drugs. Data showed that high concentration (100 μM) of Tacrolimus (*p*≥0.05) and Everolimus (*p*≥0.05) accumulated cells in G_0_/G_1_ (Fig 3D), but Sirolimus cell cycle arrest was characterized by an accumulation of cells in G_2+M_ phase (Fig 3F, *p*≥0.01). Interestingly, Tacrolimus- and Everolimus-dependent G_0_/G_1_ cell accumulation (Fig 3D) occurred as a consequence of drastic reduction in S (Fig 3E, *p*≥0.001), as well as in S (Fig 3E, *p*≥0.05) and G_2+M_ phase (Fig 3F, *p*≥0.001), respectively. The accumulation of cells in G_2+M_ phase (Fig 3F, *p*≥0.01) was related to a reduction in the percentage of cells at S phase in Sirolimus-treated HepG2 cells (Fig 3E, *p*≥0.05).

### Regulation of tumor growth, angiogenesis, cell proliferation and apoptosis by immunosuppressants in Huh 7-developed tumors in nude mice

**Fig 4:**
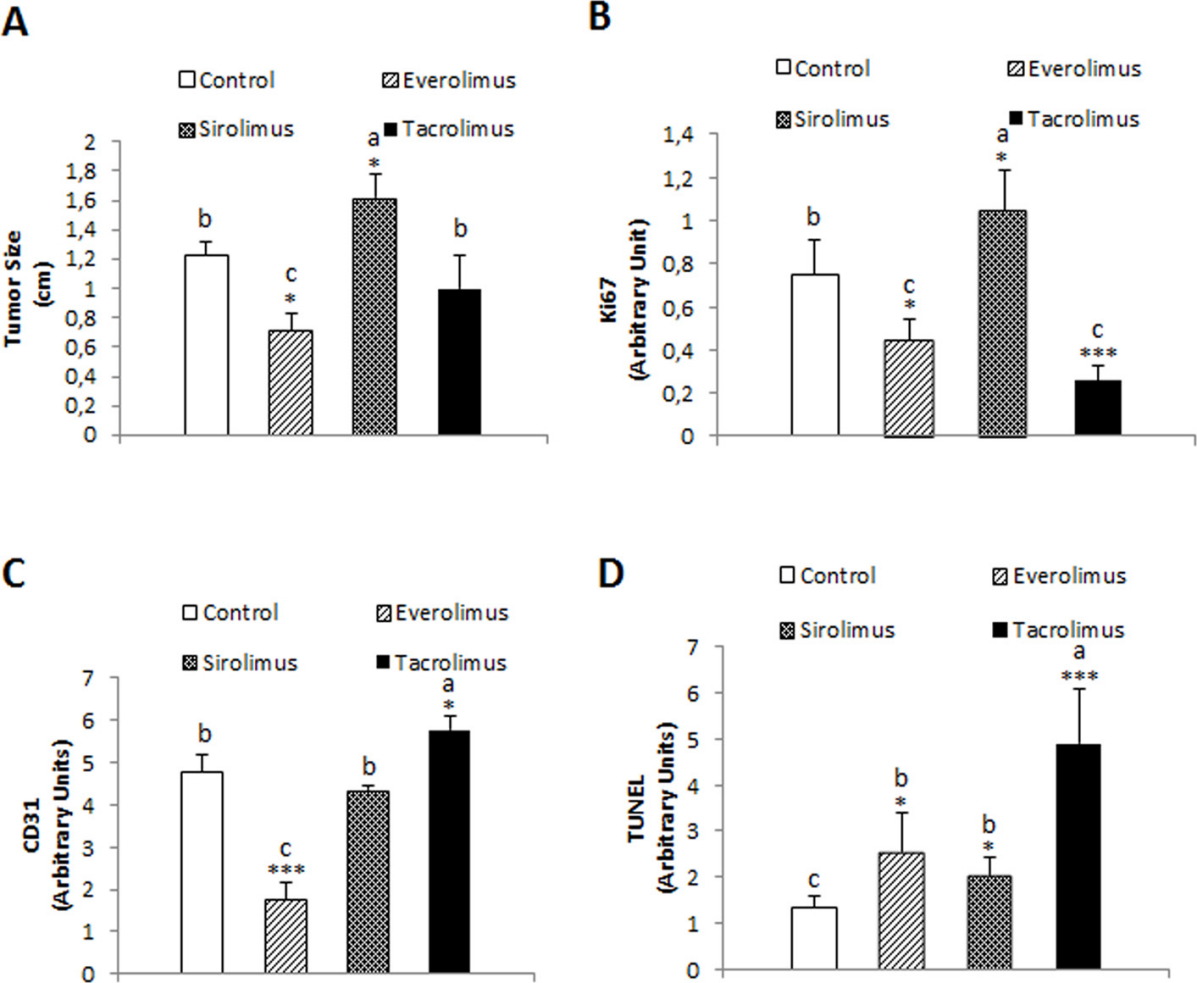
Effect of immunosuppressants on the tumor size (A), Ki67 (B), CD31 (C) and TUNEL staining (D) in tumors derived from Huh 7 cells implanted in nude mice. Cells (10x10^6^) were implanted at dorsal level in athymic mice, and 24 hours the treatments were initiated using oral administration following the dosage described in Material and Methods. Animals were sacrificed when one tumor size reached 15 mm. CD31, Ki67 and TUNEL staining were determined by immunohistochemical analysis following the procedures described in Material and Methods. Data are expressed as mean ± SEM. The groups with symbol are statistically different (*p≤0.05, **p≤0.01 and ***p≤0.001) compared with their corresponding control. The groups with different letter (a, b or c) were significantly different (*p* ≤ 0.05) compared to other groups within the same drug concentration.

**Fig 5:**
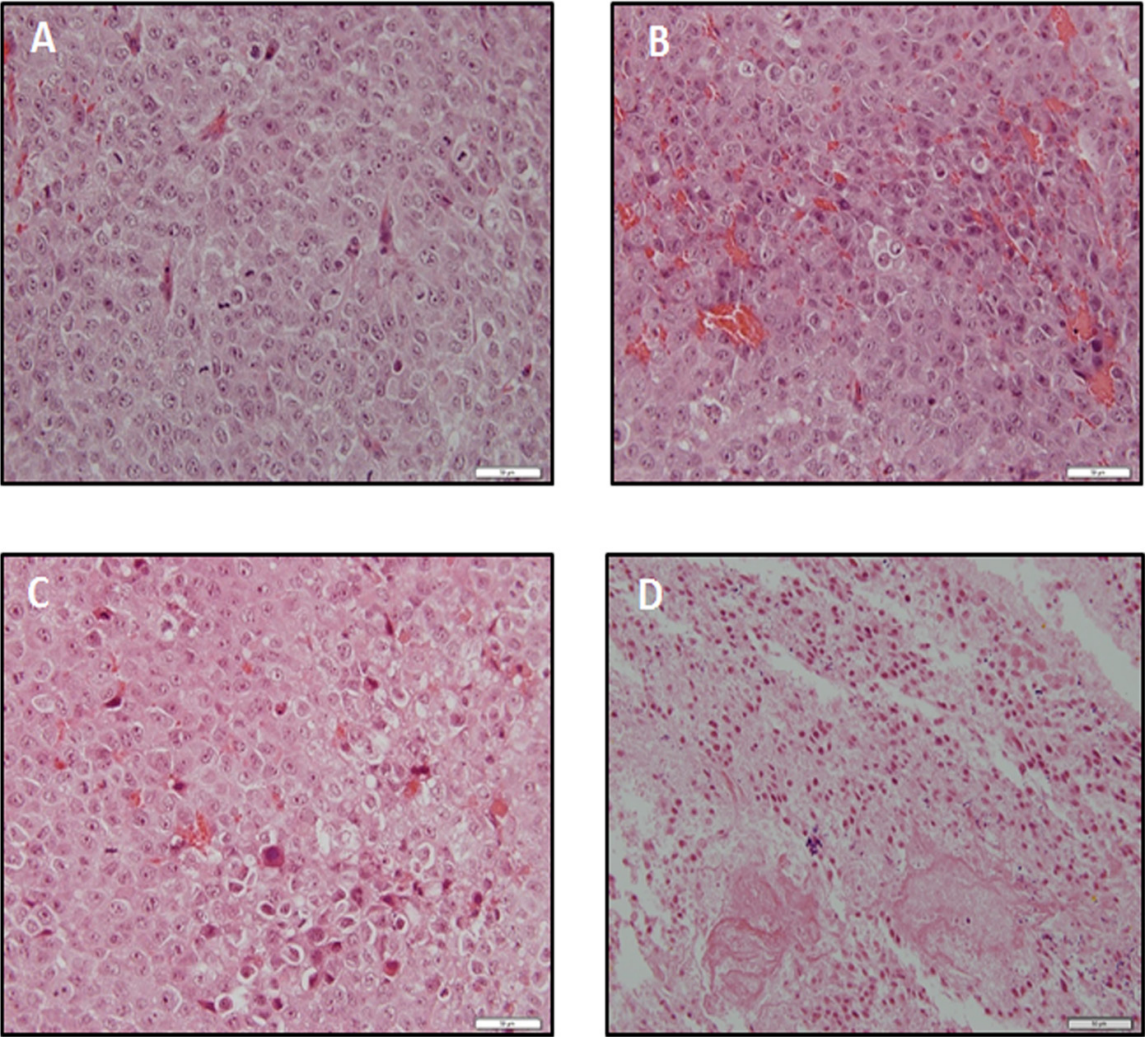
Hematoxylin-eosin staining from Control (A), Everolimus (B), Sirolimus (C) and Tacrolimus (D) tumors derived from Huh 7 cells implanted in athymic mice. Cells (10x10^6^) were implanted at dorsal level in athymic mice, and 24 hours the treatments were initiated using oral administration following the dosage described in Material and Methods. Animals were sacrificed when one tumor size reached 15 mm. The images are representative of four independent experiments. Original magnification 40x.

We assessed the antitumoral properties of Tacrolimus and mTOR inhibitors in xenograft mouse model based on the subcutaneous implantation of Huh 7 cells. Everolimus reduced significantly tumor size compared to control and Tacrolimus-treated animals (Fig 4A, *p*≥0.05). Sirolimus increased significantly tumor size compared to control group (Fig 4A, *p*≥0.05). The expression of ki67 was used as a marker of cell proliferation (Fig 4B). Everolimus (*p*≥0.05) and Tacrolimus (*p*≥0.001) reduced significantly the expression of ki67 in tumor sections while increased in Sirolimus-treated animals (Fig 4B, *p*≥0.05). We assessed the effect of immunosuppressant on angiogenesis. Interestingly, Everolimus strongly reduced the expression of CD31 in tumor sections suggesting that the drug exerted potent anti-angiogenic properties (Fig 4C, *p*≥0.001). This observation was in clear contrast to the increased expression of CD31 observed in tumor sections from Tacrolimus-treated animals (Fig 4C, *p*≥0.05). A more potent TUNEL staining was observed in tumor sections from Tacrolimus-treated animals (*p*≥0.001) than Everolimus- and Sirolimus-treated animals (Fig 4D, *p*≥0.05). The induction of tissue injury induced by treatments is observed in hematoxylin-eosin-stained tumor sections (Fig 5).

### Nephrotoxicity induced by Tacrolimus in xenograft mice model

**Fig 6:**
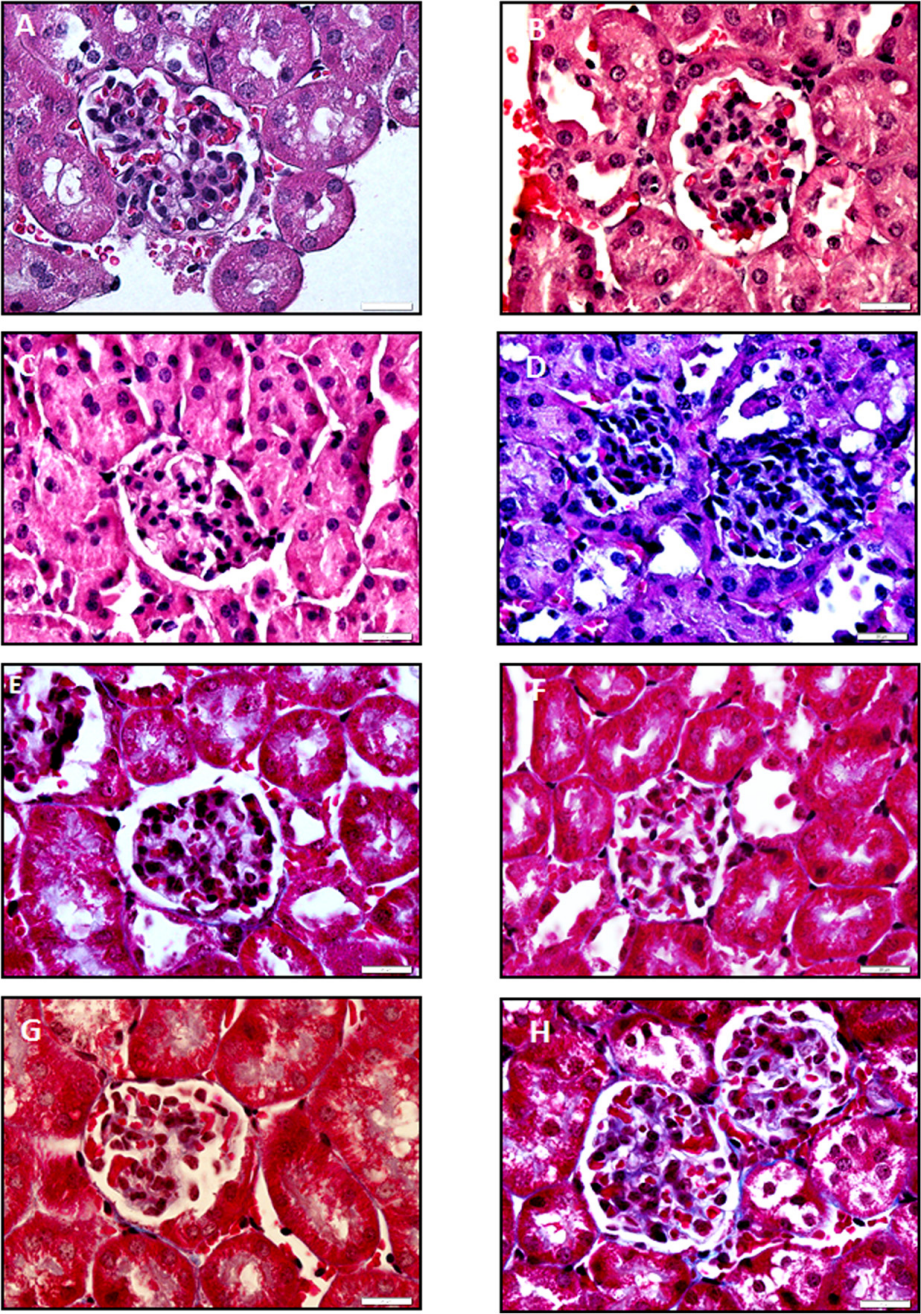
Effect of immunosuppressants on the histological structure using hematoxylin-eosin (A-D) and Masson (E-H) staining in glomerulus obtained from xenograft mice model. Kidney was removed from Huh 7 cells implanted in athymic mice. Cells (10x106) were implanted at dorsal level in athymic mice, and 24 hours the treatments were initiated using oral administration following the dosage described in Material and Methods. Control: A and E, Everolimus: B and F, Sirolimus: C and G, and Tacrolimus: D and H. Animals were sacrificed when one tumor size reached 15 mm. Hematoxylin-eosin and Masson staining were carried out using standard procedures. The images are representative of four independent experiments. Original magnification 60x.

**Fig 7:**
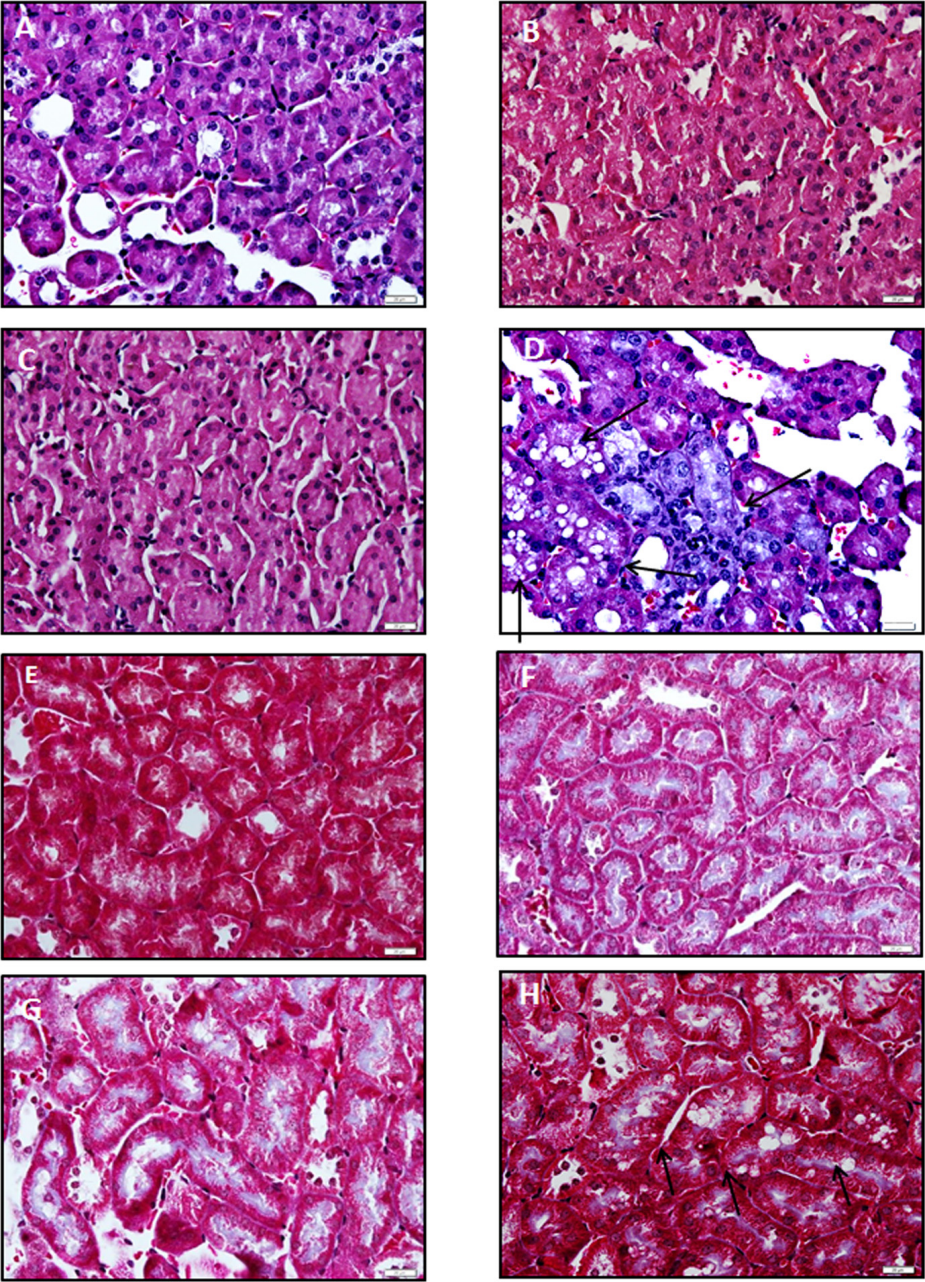
Effect of immunosuppressants on the histological structure using hematoxylin-eosin (A-D) and Masson (E-H) staining in renal tubule obtained from xenograft mice model. Kidney was removed from Huh 7 cells implanted in athymic mice. Cells (10x10^6^) were implanted at dorsal level in athymic mice, and 24 hours the treatments were initiated using oral administration following the dosage described in Material and Methods. Control: A and E, Everolimus: B and F, Sirolimus: C and G, and Tacrolimus: D and H. Animals were sacrificed when one tumor size reached 15 mm. Hematoxylin-eosin and Masson staining were carried out using standard procedures. The vacuolization induced by Tacrolimus is indicated using arrows. The images are representative of four independent experiments. Original magnification 60x.

The histological alterations in glomerulus and renal tubule by drugs were assessed in hematoxylin-eosin renal stained sections. A remarkable hyperplasia of epithelial cells and hemorrhage were observed in glomerulus sections in Tacrolimus-treated animals in comparison with control group (Fig 6D vs 6A). These histological disturbances were associated with increased fibrosis in glomerulus in Masson stained sections (Fig 6H vs 6E). No relevant signs of histological alterations were observed in glomerulus sections from mTOR inhibitor-treated animals. The vacuolization was dramatically increased in renal tubule sections stained with hematoxylin-eosin in Tacrolimus-treated animals compared to control group (Fig 7D vs 7A). These histological disturbances were also associated with increased fibrosis in renal tubule Masson stained sections (Fig 7H vs 7E).

### Regulation of angiogenesis, fibrogenesis, cell proliferation and apoptosis by immunosuppressants in kidney tissue

**Fig 8:**
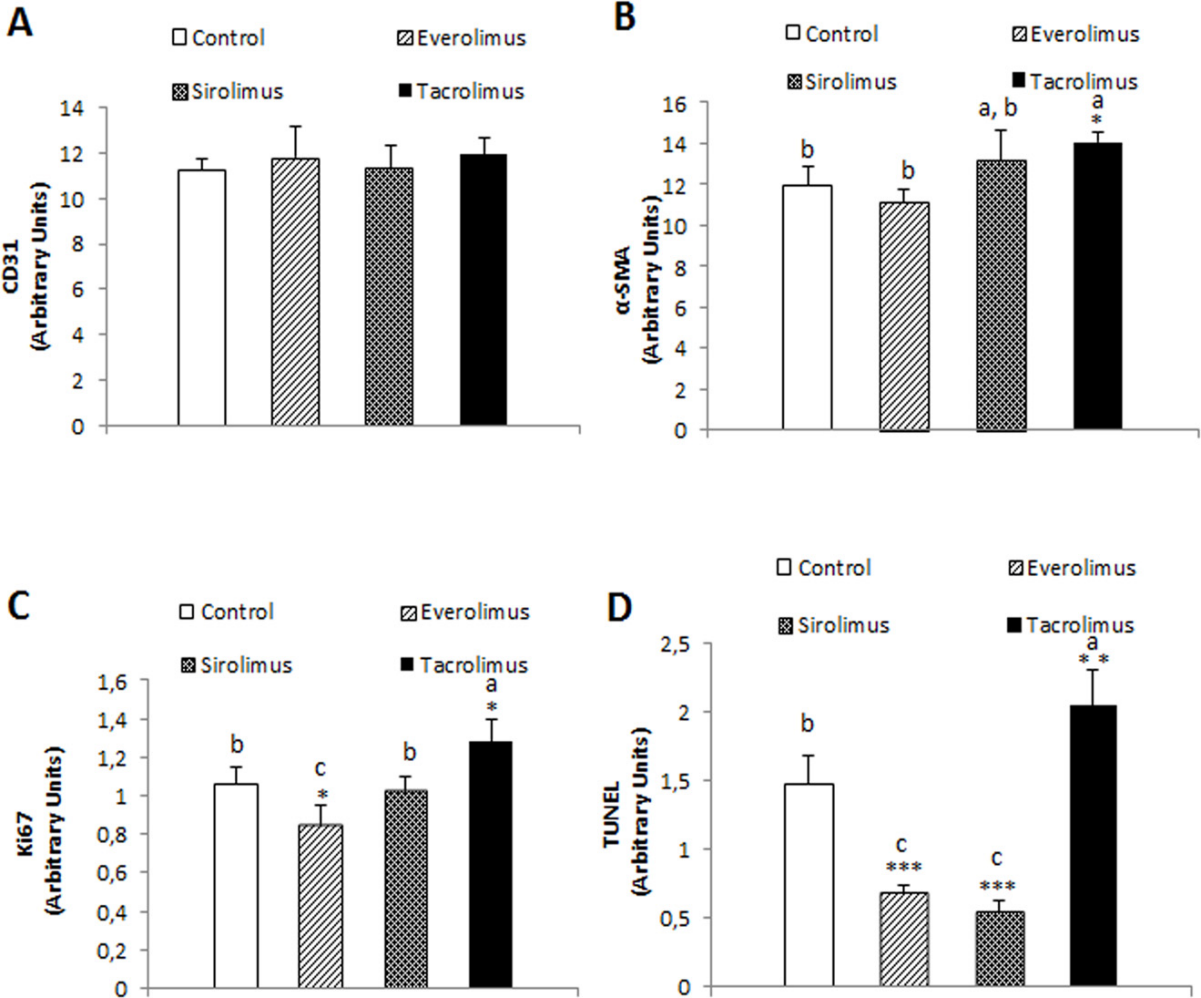
Effect of immunosuppressants on the CD31 (A), alpha-SMA (B), Ki67 (C) and TUNEL (D) in kidneys derived from Huh 7 cells implanted in athymic mice. Cells (10x10^6^) were implanted at dorsal level in athymic mice, and 24 hours the treatments were initiated using oral administration following the dosage described in Material and Methods. Animals were sacrificed when one tumor size reached 15 mm. CD31, alpha-SMA, Ki67 and TUNEL expression were determined by immunohistochemical analysis following the procedures described in Material and Methods. Data are expressed as mean ± SEM. The groups with symbol are statistically different (*p≤0.05, **p≤0.01 and ***p≤0.001) compared with their corresponding control. The groups with different letter (a, b or c) were significantly different (*p* ≤ 0.05) compared to other groups within the same drug concentration.

We evaluated the histological alterations induced by immunosuppressants in renal tissue from nude mice developing Huh 7-derived tumors. The treatments did not exert any significant effect on the expression of CD31 in renal tissue (Fig 8A). The degree of fibrogenesis (Fig 8B, *p*≥0.05), cell proliferation (Fig 8C, *p*≥0.05) and TUNEL staining (Fig 8D, *p*≥0.01) in renal tissue was significantly increased by Tacrolimus in comparison with control group. Interestingly, the expression of ki67 (Fig 8C, *p*≥0.05) and TUNEL (Fig 8D, *p*≥0.001) was significantly reduced by Everolimus in renal tissue.

## Discussion

Therapeutic immunosuppression is needed to avoid rejection and graft loss in transplantation. CNIs are currently the master immunosuppression-based protocols in OLT. Tacrolimus results in less acute rejection and better graft and patient survival when compared with Cyclosporine [19]. However, there is growing evidence suggesting that patients may be overimmunosuppressed within the first month after OLT while adverse effects such as renal toxicity is increased [20]. mTOR inhibitors (Sirolimus and Everolimus) are being increasingly used specially in OLT for the treatment of HCC due to its antiproliferative effects [15, 21], but only after 1-month post-LT, given the potential deleterious effect of the drug on surgical wound healing and hepatic artery thrombosis [22]. The resulting unbalance between cell cycle regulation and cell death may reflect the potential antitumoral properties of immunosuppressant treatments.

The present study showed that apoptosis, measured as caspase-3 activation and TUNEL staining, was only detected at the highest drug concentration (100 μM), but with significant differences in terms of pro-apoptotic potency (tacrolimus>everolimus>sirolimus) and cell line susceptibility (Huh 7>Hep3B>HepG2) (Fig 2). Tacrolimus (10 μM) has been shown to induce caspase-3 and G_0_/G_1_ cell cycle arrest in Jurkat human T lymphocytes [23]. However, other studies could not detect induction of apoptosis, measured as the percentage of hypodiploid cells in flow cytometry analysis, by Tacrolimus administered at similar concentration range in SMMC-7221 [24], Hep3B and SK-hep1 cells [25]. Although flow cytometry assay is a relevant methodological approach to identify alterations in the cell cycle profile, but it is less sensitive than measuring caspase activity. In a prospective study with 493 consecutive OLT patients under Tacrolimus regime, patients with blood trough concentrations ranging 7–10 ng/ml had longer graft survival than blood trough concentrations ranging 10–15 ng/ml [13]. In consequence, the exposure to CNI within the first month after OLT (defined as mean Tacrolimus trough concentrations >10 ng/ml) had increased 1- and 5-years HCC recurrence rates [13]. In our *in vitro* conditions, the corresponding drug concentration was located around 10–100 nM concentration which was related to transient stimulation of cell proliferation by Tacrolimus in Hep3B cells (100 nM-1μM). The induction of apoptosis was only observed at the highest Tacrolimus concentration (100 μM or 80.4 μg/ml) that was over Tacrolimus therapeutic range. No significant elevation of caspase-3 was observed below this concentration as consequence of immunosuppressant treatments.

Different cell proliferation profiles were observed during Tacrolimus and mTOR inhibitor treatments. The inhibition of mTOR by Everolimus and Sirolimus downregulates the translation of mRNA encoding essential cell cycle regulatory proteins involved in the G_1_ to S phase transition in the cell cycle [8]. The inhibition of IL-2-induced transcription by CNIs affects the expression of the proliferating cell nuclear antigen (PCNA) which functions in both DNA replication interacting with the kinase activity of cdk4/cyclin D and cdk2/cyclin E complexes, as well as repairing mechanism as a subunit of DNA polymerase δ [26]. Baksh et al. [27] showed specific binding between cdk4 and the Ca^2+^/calmodulin activated serine/threonine phosphatase or calcineurin. The study showed that the inhibition of calcineurin-dependent phosphatase by Tacrolimus and Cyclosporine increased cdk4-related activity and cell cycle progression in Jurkat cells [27]. In concordance, we have observed a significant transient increase of BrdU incorporation at intermediate concentration range (100 nM-1 μM) in Tacrolimus-treated Hep3B (Fig 3C). A similar pro-proliferative effect of Tacrolimus has also been previously observed using flow cytometry analysis in Hep3B cells [25]. This effect was not observed in HepG2 and Huh 7 cells. The absence of p53 gene expression of Hep3B in comparison with wild type p53 (HepG2) and mutated p53 (Huh 7) might be involved in the induction of cell proliferation in Tacrolimus-treated Hep3B. The administration of Tacrolimus has been shown to increase the expression of p53, p21 and p27 in urothelial cancer cells [28]. The induction of p21 expression by Tacrolimus is through transforming growth factor (TGF)-beta increased expression in human lung adenocarcinoma cells [29].

The anti-proliferative properties of the drugs were observed at the highest immunosuppressant concentration (100 μM) with similar significant values of potency in Everolimus- and Tacrolimus-treated Huh 7 and Hep3B in which cells Sirolimus was not effective (Fig 3B and 3C). The measurement of the percentage of cells in each cell cycle phase highlights specific regulation in Everolimus- and Tacrolimus-related antiproliferative properties. The administration of Everolimus and Tacrolimus induced an accumulation of cells in G_0_/G_1_ phase in HepG2 (Fig 3D) which was related to a reduction of S (Fig 3E) and G_2+M_ (Fig 3F) phases, as well as a potent reduction in the S phase in Everolimus- and Tacrolimus-treated HepG2 cells respectively, which suggest an earlier and specific blockage of cell cycle progression by CNI treatment. The previously model of cell-cycle control based on specific subsets of CDK-cyclin regulating each phase has been replaced by the minimal threshold model of cell-cycle control in which either CDK1 or CDK2 bound to cyclin A is sufficient to control interphase, whereas CDK1-cyclin B is essential to take cells into mitosis. The differences between interphase and mitotic CDKs are not necessarily due to substrate specificity, but are more likely to be a result of different localization, such as cyclins A and E are located in the nucleus and cyclin B resides in the cytoplasm, as well as a higher activity threshold for mitosis than interphase [30]. In the model, the entry of cells into S phase relies in the new cyclin synthesis, and it is relevant to notice that Tacrolimus drastically reduces the expression of cyclin D1, cyclin D3, and cyclin E in urothelial cancer cells [28]. Differently, although Everolimus (1.5 μM) induced G_0_/G_1_ cell cycle arrest based on reduced PCNA expression, higher doses (16 μM) increased cyclin D1 expression, no evidence of an accumulation of cells in G_0_/G_1_ while increasing apoptosis in pre-B acute lymphoblastic leukemia cells [31]. In addition, Everolimus (10 nM) also increased cyclin D and E expression in breast cancer MDA-MB-231 cells [32]. Huynh et al [33] also showed that the reduction of tumor growth by Everolimus was associated with the reduced expression of cyclin B, but not cyclin D, in a xenograft mouse model.

The oral administration of immunosuppressants exerted differential effect on the growth of tumors developed upon Huh 7 cell implantation in nude mice. The administration of Everolimus, but not Tacrolimus, reduced significantly tumor size (Fig 4A). However, both treatments reduced the expression of ki67 (Fig 4B) and increased TUNEL staining (Fig 3D) in tumor sections. Surprisingly, the administration of Sirolimus significantly increased tumor size (Fig 4A) and ki67 expression (Fig 4B) in tumors derived from Huh 7 cells in nude mice. Huynh et al. [34] observed a reduction of the tumor growth by Everolimus using sorafenib-less-sensitive 10–0505 xenograft developed in severe combined immunodeficiency disease (SCID) mice. The increase of tumor size induced by Sirolimus is intriguing but it correlated to an increase on ki67 expression in Huh 7-developed tumors in nude mice. The antiproliferative properties of Everolimus were associated with a reduced CD31 expression in tumors which was used as a marker of angiogenesis (Fig 4C). Interestingly, Tacrolimus increased significantly angiogenesis which is a high risk event in tumor development (Fig 4C). In fact, FKBP 12 has been shown to inhibit TGF-beta type I receptor signaling [35]. In this sense, and in concordance with our data, Giordano et al. [36] demonstrated that Tacrolimus administration activates TGF-beta-dependent cell proliferation in vascular smooth muscle cell. The simultaneous increased ki67 expression (Fig 4B) and reduction of TUNEL staining (Fig 4D) reflected the complexity of Tacrolimus signaling in tumor environment. The histological analysis using hematoxylin-eosin staining showed a remarkable induction of apoptosis upon treatments (Fig 5). Interestingly, tumor cells from Tacrolimus-treated animals showed a reduced size surrounded by extensive interstitial tissue (Fig 5D).

The study also addressed the differential histological alteration in glomerulus and renal tubule as a consequence of the in vivo administration of therapeutic doses of Tacrolimus and mTOR inhibitors. Everolimus and Sirolimus do not induce any significant histological alterations in kidney. However, hematoxylin-eosin staining showed extensive hemorrhage and epithelial hyperplasia in glomerulus (Fig 6D), as well as extensive vacuolization and necrosis in renal tubule (Fig 7D) from Tacrolimus-treated mice. The observed histological injury induced by Tacrolimus was associated with an increased ki67 (Fig 8C) and TUNEL staining (Fig 8D) in kidney sections. It was also interesting to notice, the profibrotic stimulation induced by Tacrolimus in the experimental model. In this sense, tissue sections stained with Masson showed a significant collagen deposition in glomerulus (Fig 6H) and at less extent in renal tubule (Fig 7H). The increased fibrogenic activity induced by Tacrolimus was related to increased expression alpha-SMA (Fig 8B) in kidney sections. These data are in agreement with the extensive literature showing that mTOR inhibitors do not induce nephrotoxicity compared with the Tacrolimus administration which requires lowering dosage or conversion to mTOR inhibitors [8].

In conclusion, the study showed remarkable differences between Tacrolimus and mTOR inhibitors regarding pro-apoptotic and anti-proliferative properties suggesting a role of p53 in this matter which require further studies. The pro-angiogenic properties of Tacrolimus may be an additional underlying mechanism of a risk event for tumor development. Our study, confirm and included additional information on the increased nephrotoxicity induced by Tacrolimus vs mTOR inhibitors.

## Abbreviations

CNI: Calcineurin inhibitors
CREB: cAMP response element-binding protein
FKBP: FK506-binding protein
HCC: Hepatocarcinoma
mTOR: Mammalian target of rapamycin
NFAT: Nuclear factor of activated T cells
OLT: Ortothopic liver transplant
